# A Computed Tomography-Based Radiomic Prognostic Marker of Advanced High-Grade Serous Ovarian Cancer Recurrence: A Multicenter Study

**DOI:** 10.3389/fonc.2019.00255

**Published:** 2019-04-09

**Authors:** Wei Wei, Zhenyu Liu, Yu Rong, Bin Zhou, Yan Bai, Wei Wei, Shuo Wang, Meiyun Wang, Yingkun Guo, Jie Tian

**Affiliations:** ^1^Engineering Research Center of Molecular and Neuro Imaging of Ministry of Education, School of Life Science and Technology, Xidian University, Xi'an, China; ^2^CAS Key Laboratory of Molecular Imaging, Institute of Automation, Chinese Academy of Sciences, Beijing, China; ^3^School of Applied Technology, Xi'an Polytechnic University, Xi'an, China; ^4^School of Artificial Intelligence, University of Chinese Academy of Sciences, Beijing, China; ^5^Key Laboratory of Intelligent Medical Image Analysis and Precision Diagnosis in Guizhou Province, Department of Radiology, Guizhou Provincial People's Hospital, Guiyang, China; ^6^Key Laboratory of Birth Defects and Related Diseases of Women and Children of Ministry of Education, West China Second University Hospital, Sichuan University, Chengdu, China; ^7^Department of Radiology, Henan Provincial People's Hospital, Zhengzhou, China; ^8^Key Laboratory of Birth Defects and Related Diseases of Women and Children of Ministry of Education, Department of Radiology, West China Second University Hospital, Sichuan University, Chengdu, China; ^9^Beijing Advanced Innovation Center for Big Data-Based Precision Medicine, School of Medicine, Beihang University, Beijing, China

**Keywords:** advanced high-grade serous ovarian cancer, CT, prognosis, radiomics, recurrence

## Abstract

**Objectives:** We used radiomic analysis to establish a radiomic signature based on preoperative contrast enhanced computed tomography (CT) and explore its effectiveness as a novel recurrence risk prognostic marker for advanced high-grade serous ovarian cancer (HGSOC).

**Methods:** This study had a retrospective multicenter (two hospitals in China) design and a radiomic analysis was performed using contrast enhanced CT in advanced HGSOC (FIGO stage III or IV) patients. We used a minimum 18-month follow-up period for all patients (median 38.8 months, range 18.8–81.8 months). All patients were divided into three cohorts according to the timing of their surgery and hospital stay: training cohort (TC) and internal validation cohort (IVC) were from one hospital, and independent external validation cohort (IEVC) was from another hospital. A total of 620 3-D radiomic features were extracted and a Lasso-Cox regression was used for feature dimension reduction and determination of radiomic signature. Finally, we combined the radiomic signature with seven common clinical variables to develop a novel nomogram using a multivariable Cox proportional hazards model.

**Results:** A final 142 advanced HGSOC patients were enrolled. Patients were successfully divided into two groups with statistically significant differences based on radiomic signature, consisting of four radiomic features (log-rank test *P* = 0.001, <0.001, <0.001 for TC, IVC, and IEVC, respectively). The discrimination accuracies of radiomic signature for predicting recurrence risk within 18 months were 82.4% (95% CI, 77.8–87.0%), 77.3% (95% CI, 74.4–80.2%), and 79.7% (95% CI, 73.8–85.6%) for TC, IVC, and IEVC, respectively. Further, the discrimination accuracies of radiomic signature for predicting recurrence risk within 3 years were 83.4% (95% CI, 77.3–89.6%), 82.0% (95% CI, 78.9–85.1%), and 70.0% (95% CI, 63.6–76.4%) for TC, IVC, and IEVC, respectively. Finally, the accuracy of radiomic nomogram for predicting 18-month and 3-year recurrence risks were 84.1% (95% CI, 80.5–87.7%) and 88.9% (95% CI, 85.8–92.5%), respectively.

**Conclusions:** Radiomic signature and radiomic nomogram may be low-cost, non-invasive means for successfully predicting risk for postoperative advanced HGSOC recurrence before or during the perioperative period. Radiomic signature is a potential prognostic marker that may allow for individualized evaluation of patients with advanced HGSOC.

## Introduction

Ovarian cancer is the leading cause of gynecological cancer-related deaths (1). Seventy percent of these deaths are due to high-grade serous ovarian cancer (HGSOC) (2, 3) while 60% of such patients are diagnosed at an advanced stage (1). Although a significant proportion of patients experience a complete clinical remission with aggressive surgery and platinum-taxane chemotherapy (4), the median progress-free survival (PFS) in advanced HGSOC patients is 18 months, with most advanced HGSOC patients with recurrence experiencing a PFS of <3 years (5–7). Therefore, predictive recurrence in advanced HGSOC patients is critical for the identification of precise, personalized treatment, and follow-up plans that prolong patient survival. Currently, predicting the recurrence of advanced HGSOC during the perioperative period remains limited. Development of prognostic markers of advanced HGSOC are thus critical to improving outcomes in these patients.

Contrast enhanced computed tomography (CT), a routinely used diagnostic tool, provides a non-invasive and low-cost method for extracting HGSOC prognostic information (8). Radiomics, a subset of the field of medical imaging research, has progressed dramatically in recent years, enabling comprehensive expression of tumor heterogeneity and more advanced prognostic applications (9). Using the high-throughput quantitative radiomic features often extracted from medical images, clinicians can develop personalized treatment plans and improve tumor detection strategies, as well as phenotypically subtype and evaluate the curative effects of particular treatments as well as patients' prognoses (10–12). Radiomics approach is an effective tool for exploring the relationships among radiomic features and patients' prognoses, which may promote new ideas and improvements for oncological decision-support (13). In particular, radiomics has been successfully applied to determining tumor prognosis (14) and HGOSC recurrence (15, 16).

In this retrospective multicenter study, we hypothesized that radiomic analysis would provide a prognostic marker (radiomic signature) of advanced HGSOC recurrence. We performed a radiomic analysis to extract CT-based quantitative radiomic features and developed a novel prognostic marker (radiomic signature) for individualized, pretreatment evaluation of PFS in patients with advanced HGSOC. Furthermore, we validated the predictive ability of this radiomic signature over 18 months and 3 years, respectively. Moreover, we developed a novel nomogram in conjunction with this radiomic signature and revealed seven common clinical characteristics that might be associated with relapse. Collectively, these findings provide potentially critical insights into individualized treatment and follow-up planning.

## Materials and Methods

We enrolled 142 patients with advanced HGSOC. All patients were enrolled between March 2010 and September 2015 at West China Second University Hospital of Sichuan University, Chengdu, China (WCSUH-SCU), or between May 2012 and October 2016 at Henan Provincial People's Hospital, Zhengzhou, China (HNPPH). The ethics committee of WCSUH-SCU and HNPPH approved this study and the requirement for informed consent was waived. Our study was conducted in accordance with the Declaration of Helsinki.

### Eligibility Criteria

**Figure 1:**
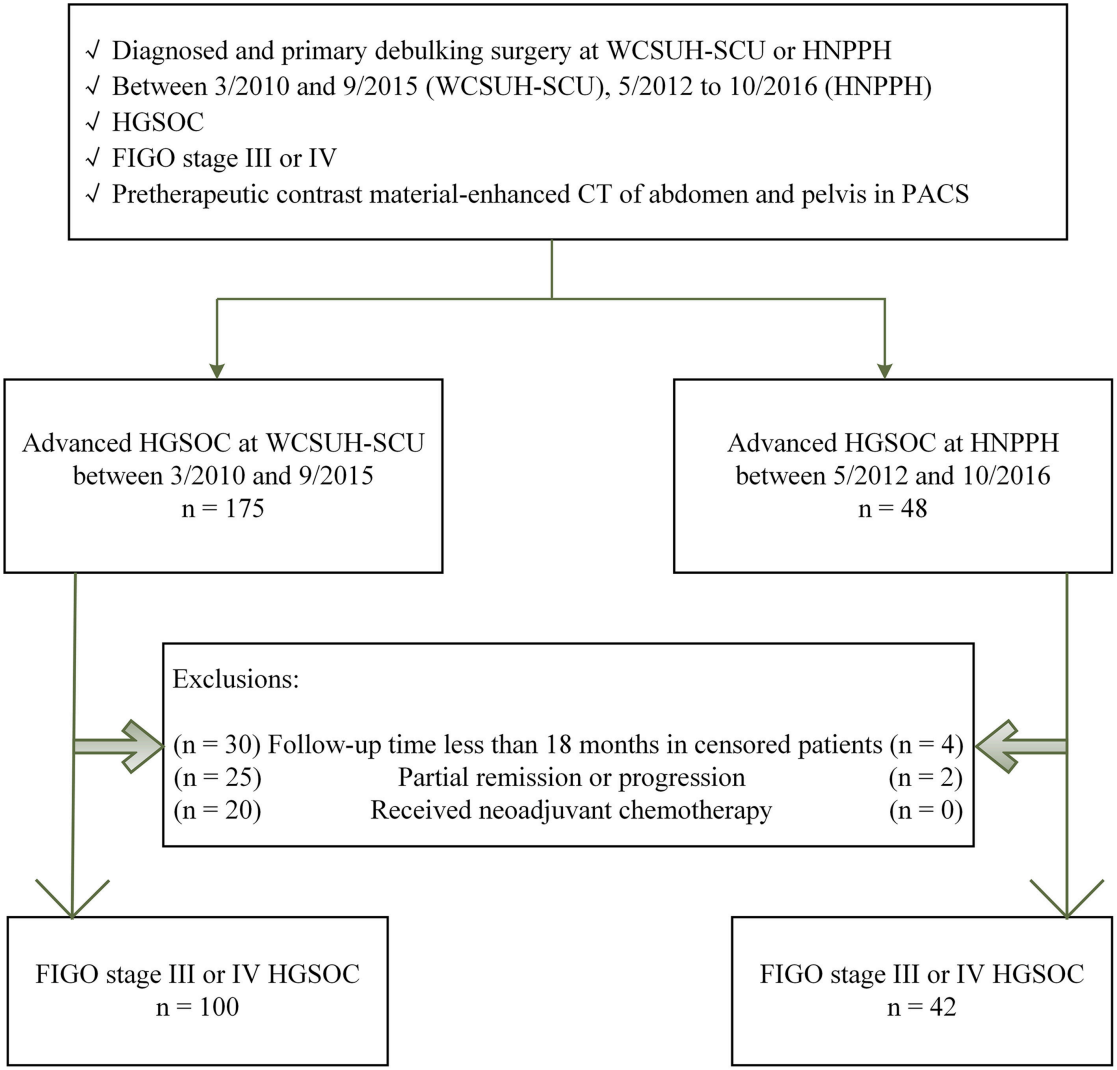
Eligibility criteria. Flowchart depicting the patient selection process. WCSUH-SCU, West China Second University Hospital of Sichuan University; HNPPH, Henan Provincial People's Hospital; HGSOC, High-Grade Serous Ovarian Cancer; FIGO, International Federation of Gynecology and Obstetrics; PACS, Picture Archiving and Communication System.

Patient inclusion criteria were: (a) pathologically confirmed International Federation of Gynecology and Obstetrics (FIGO) stage III or IV HGSOC, (b) diagnosis made at and primary debulking surgery (PDS) performed at WCSUH-SCU or HNPPN, (c) preoperative contrast enhanced CT of the abdomen and pelvis via the Picture Archiving and Communication System (PACS), and (d) available follow-up data. Patient exclusion criteria were: (a) a follow-up time of <18 months in censored patients, (b) a response to PDS including partial remission or progression, (c) undergoing neoadjuvant chemotherapy (NACT) followed by interval debulking surgery (IDS) as NACT alters CT findings. Details of study inclusion and exclusion criteria are summarized in Figure 1.

One hundred enrolled patients from WCSUH-SCU were divided into a training cohort (TC) and an internal validation cohort (IVC) based on their time of surgery at a 1:1 ratio. Fifty patients with an early surgical time were allocated to the TC group while another 50 patients with a late surgery time comprised the IVC group. The 42 enrolled patients from HNPPH were used as an independent external validation cohort (IEVC). In addition, we also collected clinical information from all patients including their age at surgery, preoperative carbohydrate antigen 125 (CA-125) levels, postoperative CA-125 levels, FIGO stage, residual tumor size, tumor side, and menopause status.

### Primary Treatment

Primary debulking was performed according to WCSUH-SCU or HNPPH surgical templates, including at least total abdominal hysterectomy (TAH), bilateral salpingo-oophorectomy (BSO), omentectomy, and pelvic/para-aortic lymphadenectomy. Additional resections were performed at the discretion of the surgeon. Six to eight cycles of taxane/carboplatin chemotherapy were then performed postoperatively.

### Follow-Up and Clinical Endpoints

All patients were followed every 3 months for the first 2 years, every 6 months for the following 3–5 years, and annually thereafter. The PFS was the primary end point for our study. Recurrence dates were determined according to a follow-up physical exam, CT findings, and CA-125 levels. Follow-up times were defined as the time between complete clinical remission and clinical recurrence or the time of the last follow-up.

### CT Parameters

The patients of WCSUH-SCU were examined using a multidetector CT scanner (Brilliance 6, Philips Medical System, Best, Netherlands), scanning parameters were as follows: tube voltage, 120 kVp; tube current, 230 mA; beam pitch, 0.9; reconstruction thickness, 2 mm; reconstruction interval, 1.5 mm. Contrast medium 80–100 mL (Iopamidol 370, Bracco, Italy) was injected into the antecubital vein using a mechanical injector at a rate of 2.5–3.5 mL/sec.

The patients of HNPPH were examined using a multidetector CT scanner (Brilliance 16, Philips Medical System, Best, Netherlands; GE Discover CT 750HD, GE LightSpeed VCT 64, GE Medical Systems, Milwaukee, USA), scanning parameters were as follows: tube voltage, 120 kVp; tube current, automatic milliampere setting with a range 100–500 mA; beam pitch, <1; reconstruction thickness, 2.0 (Brilliance 16) or 5.0 (GE Discover CT 750HD and GE LightSpeed VCT 64) mm; reconstruction interval, 1.2 (Brilliance 16 and GE Discover CT 750HD) or 1.25 (GE LightSpeed VCT 64) mm. Contrast medium 60–100 mL (Ultravist 370, Bayer, Germany) was injected into the antecubital vein using a mechanical injector at a rate of 3–3.5 mL/s.

CT examinations in this study were strictly performed in accordance with the principle of “As Low As Reasonably Achievable” (ALARA), and the radiation doses were recorded. During the period of examination, patient was in suspended respiration. The scan area was from the symphysis pubis to the diaphragm, including non-enhancement scan, arterial phase and venous phase. The time delay from contrast agent injection to image acquisition was approximately 70 s.

### Tumor Segmentation

We used preoperative contrast enhanced CT Digital Imaging and Communications in Medicine (DICOM) data from the PACS. All CT DICOM images were collected from four different scanners with different scanning parameters. The ITK-SNAP (www.itksnap.org) was used for 3-D manual segmentation performed by three experienced radiologists. Masks of the tumors were drawn on CT images by two board-certified radiologists with more than 8 years of experience in ovarian cancer, which were blinded to the patients' clinical information. The region of interest (ROI) covered the whole tumor and was delineated on each slice of the CT image. These masks were combined when the difference between the individual masks identified by the two radiologists was <5%. When the difference between the two masks was greater than 5%, the masks were determined by a senior radiologist with more than 20 years of experience in ovarian cancer.

Next, DICOM images and segmentation results were normalized according to pixel spacing and slice thickness. We determined the minimum pixel spacing and slice thickness parameter values for all CT images. Then processed the original image and segmentation with a linear interpolation algorithm based on these minimum values. Finally, normalized CT and tumors to the same physical space.

### Radiomic Features Extraction

Radiomic features expressing tumor characteristics were high-dimensional quantitative features extracted from CT images. In the present study, we investigated a feature-based approach to explore meaningful and reliable information associated with progress-free survival in patients with advanced high-grade serous ovarian cancer from pre-therapeutic contrast material-enhanced CT data. In total, we extracted 620 quantitative features including imaging features previous used features in 9. These features could be divided into four groups as follows (Table 1): histogram (17 features), shape (8 features), textural (51 features), and wavelet (544 features). A filtering process was performed to implement image smoothing and image difference before CT radiomic feature extraction. 3D “Coiflet 1” wavelet transform on CT images with 8 decompositions: *LLL, LLH, LHL, LHH, HLL, HLH, HHL, HHH*, considering *L* and *H* to be a low-pass (i.e., a scaling) and a high-pass (i.e., a wavelet) function. Then re-calculate the histogram and textural features. The definition of radiomic features can be found in our previous research (17).

**Table 1:** Radiomic features extracted in our study.

**Histogram (*n* = 17)**	**Shape (*n* = 8)**	**GLCM (*n* = 22)**	**GLSZM (*n* = 13)**	**GLRLM (*n* = 11)**	**NGTDM (*n* = 5)**
Energy	Compactness1	Autocorrelation	Short zone emphasis	Short run emphasis	Contrast
Entropy	Compactness2	Cluster Prominence	Large zone emphasis	Long run emphasis	Busyness
Standard Entropy	Maximum 3D diameter	Cluster Shade	Gray-level non-uniformity	Gray-level non-uniformity	Complexity
Kurtosis	Spherical disproportion	Cluster Tendency	Zone-size non-uniformity	Run-length non-uniformity	Coarseness
Maximum	Sphericity	Contrast	Zone percentage	Run percentage	Strength
Mean	Surface area	Correlation	Low gray-level zone emphasis	Low gray-level run emphasis	
Mean absolute deviation	Surface to volume ratio	Difference entropy	High gray-level zone emphasis	High gray-level run emphasis	
Median	Volume	Dissimilarity	Small zone low gray-level emphasis	Short run low gray-level emphasis	
Minimum		Energy	Small Zone High Gray-Level Emphasis	Short run high gray-level emphasis	
Mass		Entropy	Large zone low gray-level emphasis	Long run low gray-level emphasis	
Range		Homogeneity1	Large zone high gray-level emphasis	Long run high gray-level emphasis	
Root mean square		Homogeneity2	Gray-level variance		
Skewness		Information measure of correlation1	Zone-size variance		
Standard deviation		Information measure of correlation2			
Uniformity		Inverse difference moment normalized			
Standard uniformity		Inverse difference normalized			
Variance		Inverse variance			
		Maximum probability			
		Sum average			
		Sum entropy			
		Sum variance			
		Variance			

*GLCM, gray level co-occurrence matrix; GLRLM, gray-level run-length matrix; GLSZM, gray-level size zone matrix; NGTDM, neighborhood gray-tone difference matrix (17)*.

### Radiomic Features Selection and Radiomic Signature Building

Due to the extraction of high dimensional radiomic features in this study, had all 620 radiomic features were used to build radiomic signature, over-fitting would have occurred. Therefore, we used a least absolute shrinkage and selection operator (LASSO) regression to select for features which were most closely related to recurrence (18). The parameter λ was selected in LASSO through the smallest leave one out cross-validation (LOOCV) error. After L1 regularization, the coefficients for most radiomic features were reduced to zero and any remaining non-zero coefficient radiomic features were selected. Next, we built a Cox model with these select radiomic features. The radiomic signature value for each patient was a linearly-weighted combination of the features with non-zero coefficients. All radiomic feature extraction, dimensionality reductions, and radiomic signature construction algorithms were implemented using MATLAB R2016a (MathWorks, Natick, MA).

### Validation of Radiomic Signature and Development of an Individualized Prognostic Model

The potential association between radiomic signature and PFS was validated in the TC, IVC, and IEVC, respectively. Kaplan–Meier survival analysis was used in each cohort. Patients from each cohort were divided into high-risk and low-risk groups by the median radiomic signature of the TC. The relationship between radiomic signature and PFS was determined with a log-rank test. A univariable Cox proportional hazards model was used to calculate the concordance index (C-index) for the radiomic signature and to predict the individual probabilities of 3-year and 18-month PFS after complete clinical remission in each cohort. The discriminant accuracy of the univariable Cox model was evaluated using a time-dependent C-index (constructed with the nearest neighbor estimator).

A multivariable Cox proportional hazards model was constructed using the radiomic signature and seven easily available clinical characteristics that might be associated with relapse in TC. The radiomic signature, age and preoperative CA-125 were used as continuous variables, while the others were used as categorical variables. All categorical variables were dichotomized (FIGO stage, III or IV; postoperative CA-125 ≤ 35 or >35 U/mL; residual tumor, = 0 cm or >0 cm; tumor side, unilateral or bilateral; menopause status, menopause or premenopausal). We used these independent predictors to build a multivariable cox model and then developed a novel radiomic nomogram to predict the individual probabilities of 3-year and 18-month PFS after complete clinical remission (19). Then, these clinical characteristics were used to develop a clinical prognostic model. Two prognostic models were used to predict the individual probabilities of 3-year and 18-month PFS and the discriminant accuracy of the multivariate Cox models (radiomic nomogram model and clinical prognostic model) were evaluated using a time-dependent C-index (constructed with the nearest neighbor estimator). The DeLong's test (20) was used to compare the nomogram model and clinical prognostic model. The 95% confidence interval (CI) of the C-indices was calculated by bootstrapping with a 1000 resample method (21).

**Figure 2:**
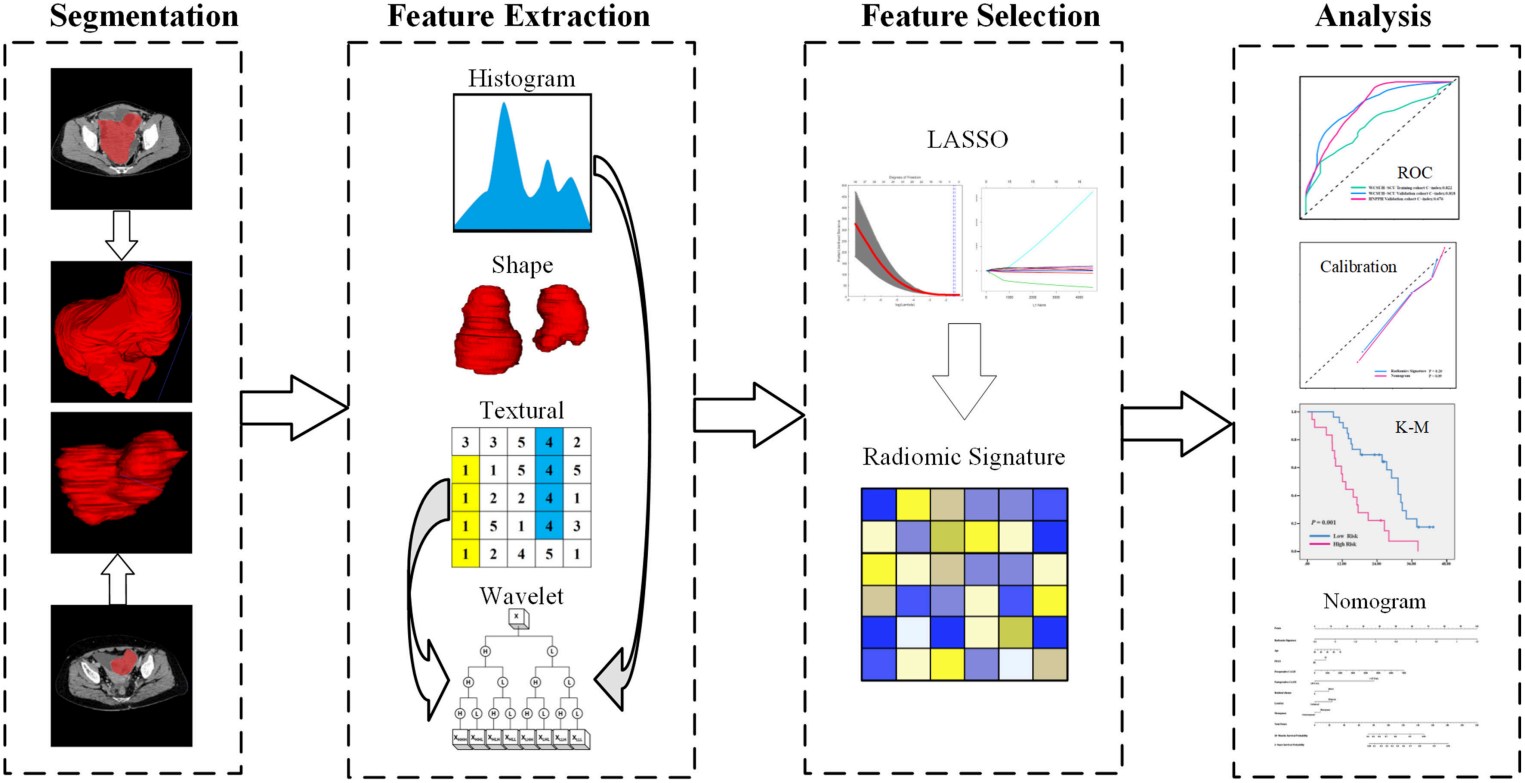
Study flowchart. LASSO, least absolute shrinkage and selection operator; ROC, receiver operating characteristic; K-M, Kaplan-Meier.

A calibration curve (22) was used to assess the degree of variability in radiomic signature and nomogram prediction and to compare their predicted recurrence probabilities with true recurrence probabilities. Each group contained at least 10 patient samples. The calibration curve was tested using the Hosmer-Lemeshow test (23) to determine whether the predicted curve and the true curve significantly differed. This study design is illustrated in Figure 2.

### Statistical Analyses

Median and interquartile ranges (IQRs) for all demographic and clinical data were reported for radiomic signature, age, and preoperative CA-125 levels. Frequencies and proportions were reported for other categorical variables. Differences in continuous variables and categorical variables were examined using the *F*-test/independent samples *t*-test and Fisher exact test, respectively. All statistical tests were two-sided. Significance was set as *P* < 0.05. Validation of radiomic signature, construction of Cox models and statistical analyses were implemented with R version 3.5.1 (R Foundation for Statistical Computing, Vienna, Austria).

## Results

### Demographic and Clinical Data

The primary clinical and pathological attributes of all 142 patients are listed in Tables 2, 3. Median (IQRs) patient age at surgery was 50 years (44.5–57 years). Median (IQRs) preoperative CA-125 levels were 713.6 U/mL (401.9–2179.8 U/mL). There were 54% patients whose postoperative CA-125 was ≤ 35 U/mL. These patients with low CA-125 levels had significantly better prognoses (*P* = 0.002). Most patients were FIGO stage III (79%), who also had a significantly lower recurrence rate (*P* = 0.021). Forty-two percent of PDS outcomes were “no gross residual.” Half of all tumors were unilateral and the other half were bilateral. Menopausal women constituted the majority of all patients (71%). Eighty-one patients (57%) had documented PFS during the study period. The median (IQRs) follow-up time was 38.8 months (32.5–45.8 months) in the censored patients. The median (IQRs) PFS was 17.9 months (13.0–26.7 months) and only 2 patients had a platinum-free interval length <6 months. The median (IQRs) number of days between obtaining CT images and undergoing surgery was 10 days (4–56 days). There were no significant statistical differences between the two cohorts in clinical variables with the exception of recurrence rate (Table 2). There were also no significant statistical differences between the recurrence and no recurrence groups with the exception of postoperative CA-125 level and FIGO stage (Table 3).

**Table 2:** Clinical patient characteristic by cohort.

**Characteristics**	**Training cohort of WCSUH-SCU** **(*n* = 50)**	**Validation cohort of WCSUH-SCU** ** (*n* = 50)**	**Validation cohort of HNPPH** ** (*n* = 42)**	***p*-value**
Age at surgery (years), median (IQRs)	50 (46–59.5)	50 (44.5–57.5)	50 (41.5–56)	0.283
Preoperative CA-125 (U/mL), median (IQRs)	913.3 (396.4–2193.6)	1346.4 (405.8–3093.6)	482.5 (402.0–713.6)	0.611
Postoperative CA-125 (%)				
≤ 35 U/mL	25 (50)	28 (56)	23 (55)	0.831
>35 U/mL	25 (50)	22 (44)	19 (45)	
FIGO stage (%)				
III	43 (86)	41 (82)	28 (67)	0.070
IV	7 (14)	9 (18)	14 (33)	
Residual (%)				
= 0	12 (24)	14 (28)	8 (19)	0.584
>0	38 (76)	36 (72)	34 (81)	
Tumor side (%)				
Unilateral	23 (46)	20 (40)	23 (55)	0.377
Bilateral	27 (54)	30 (60)	19 (45)	
Menopause status (%)				
Menopause	37 (74)	33 (66)	31 (74)	0.640
Premenopausal	13 (26)	17 (34)	11 (26)	
Recurrence (%)				
Yes	20 (40)	29 (58)	32 (76)	0.002
No	30 (60)	21 (42)	10 (24)	
Follow-up in censored patients (month), median (IQRs)	46.1 (42.9–55.7)	33.6 (31.4–35.2)	25.6 (21.0–32.4)	–
Follow-up in recurrence (month), median (IQRs)	26.6 (18.7–29.2)	16.5 (12.6–20.0)	16.4 (9.7–28.1)	–

*p-values are the result of Fisher exact tests (categorical variables) or F-tests (continuous variables). WCSUH-SCU, West China Second University Hospital of Sichuan University; HNPPH, Henan Provincial People's Hospital; CA-125, Carbohydrate Antigen 125; FIGO, International Federation of Gynecology and Obstetrics; IQRs, interquartile ranges*.

**Table 3:** Clinical characteristic of patients in recurrence and no recurrence cohorts.

**Characteristics**	**All Patients** **(*n* = 142)**	**Recurrence** ** (*n* = 81)**	**No recurrence** ** (*n* = 61)**	***p*-value**
Age at surgery (years) median (IQRs)	50 (44.5–57)	50 (45–57)	50 (43.5–57)	0.438
Preoperative CA-125 (U/mL) median (IQRs)	713.6 (401.9–2179.8)	609.7 (417.2–2127.9)	851.9 (396.2–2020.2)	0.951
Postoperative CA-125 (%)				
≤ 35 U/mL	76 (54)	34 (42)	42 (69)	0.002
>35 U/mL	66 (46)	47 (58)	19 (31)	
FIGO stage (%)				
III	112 (79)	58 (72)	54 (89)	0.021
IV	30 (21)	23 (28)	7 (11)	
Residual (%)				
= 0	60 (42)	34 (42)	26 (43)	1.000
>0	82 (58)	47 (58)	35 (57)	
Tumor side (%)				
Unilateral	71 (50)	43 (53)	28 (46)	0.498
Bilateral	71 (50)	38 (47)	33 (54)	
Menopause status (%)				
Menopause	101 (71)	59 (73)	42 (69)	0.709
Premenopausal	41 (29)	22 (27)	19 (31)	
Follow-up (month) median (IQRs)	27.7 (17.2–37.8)	17.9 (13.0–26.7)	38.8 (32.5–45.8)	–

*p-values are the result of Fisher exact tests (categorical variables) or independent-samples t-tests (continuous variables). CA-125, Carbohydrate Antigen 125; FIGO, International Federation of Gynecology and Obstetrics; IQRs, interquartile ranges*.

### Radiomic Features Selection and Radiomic Signature Building

Based on the TC, we selected four radiomic features from 620 high-dimensional features that were most strongly associated with PFS to build the radiomic signature. These included the zone-size variance in the gray-level size zone matrix (GLSZM) of textural features extracted from the Coiflet_LLL_ wavelet transform, the first-order statistics (FOS) feature, which describes the maximum intensity value extracted from the Coiflet_LHL_ wavelet transform, the FOS feature, which describes the maximum value of the intensity levels extracted from the Coiflet_LHH_ wavelet transform, and the small zone low gray-level emphasis in the GLSZM of textural features extracted from the Coiflet_HLL_ wavelet transform. The details of selected four radiomic features are described in Table 4.

**Table 4:** Four radiomic features selected by LASSO-Cox.

**Radiomic features**	**Coefficients of LASSO-Cox**	**C-index (95% CI)**	***P-*value**
Coiflet_LLL_ GLSZM ZSV	5.47648232895881e-06	0.624 (0.565–0.684)	0.036
Coiflet_LHL_ FOS maximum	−0.0178879313170910	0.673 (0.604–0.743)	0.012
Coiflet_LHH_ FOS maximum	−0.0122131044045091	0.669 (0.608–0.731)	0.001
Coiflet_HLL_ GLSZM SZLGE	−229.560623168945	0.552 (0.492–0.612)	0.388

*LASSO, least absolute shrinkage and selection operator; GLSZM, gray-level size zone matrix; ZSV, zone-size variance; FOS, first-order statistics; SZLGE, small zone low gray-level emphasis*.

### Validation of Radiomic Signature and Prognostic Model

**Figure 3:**
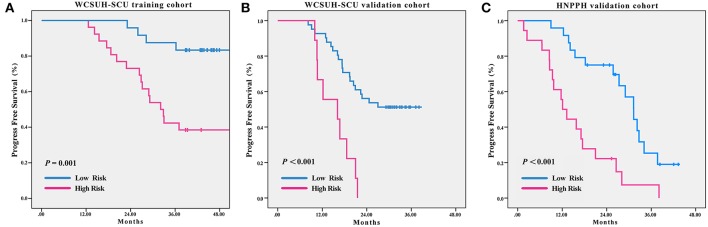
Clinical recurrence-free survival stratified by risk according to radiomic signature. Kaplan-Meier curves showing clinical recurrence-free survival in patients stratified by radiomic signature risk and classification in the WCSUH-SCU training cohort **(A)**, the WCSUH-SCU internal validation cohort **(B)**, and the HNPPH independent external validation cohort **(C)**. High-risk and low-risk curves were compared with the log-rank test. WCSUH-SCU, West China Second University Hospital of Sichuan University; HNPPH, Henan Provincial People's Hospital.

**Figure 4:**
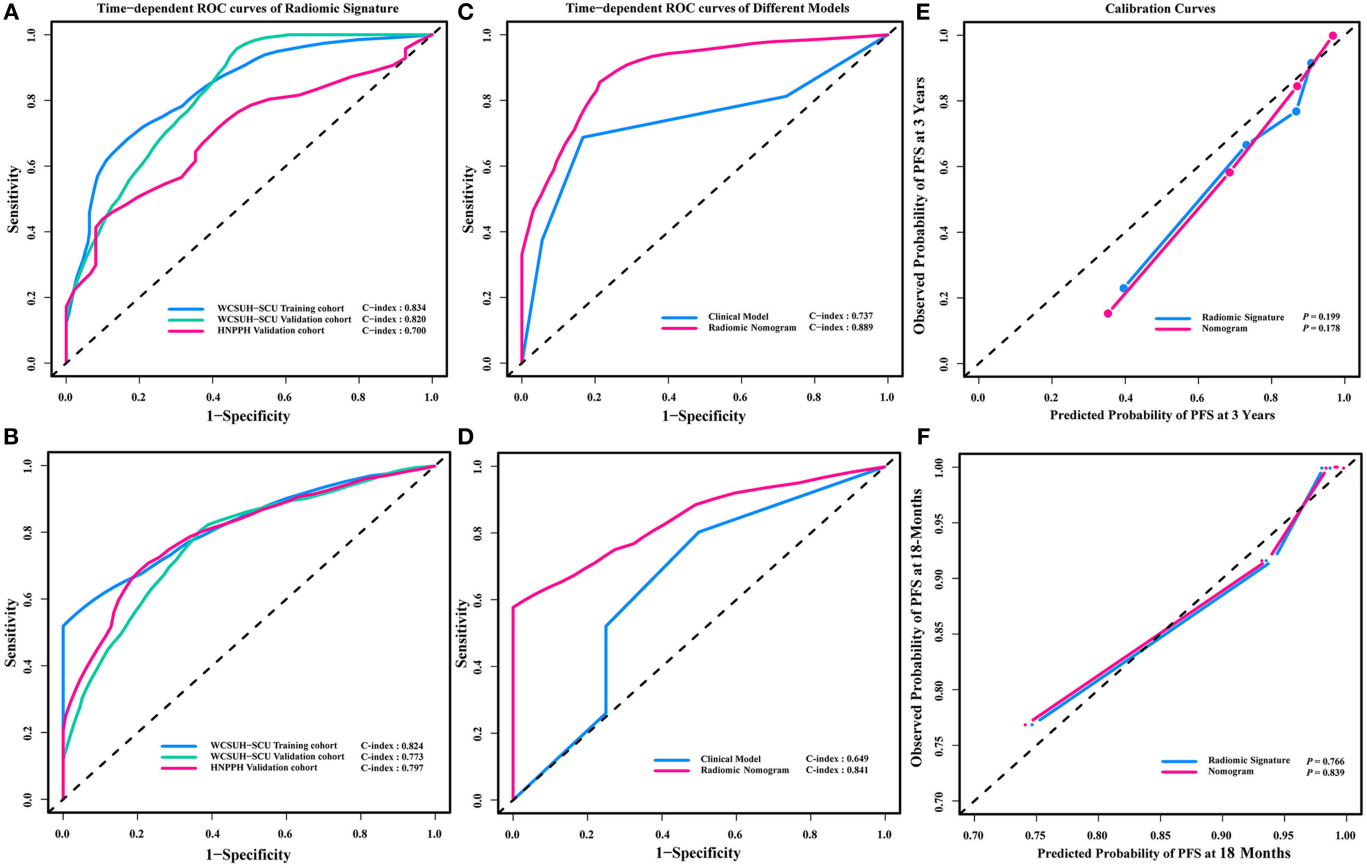
Time-dependent ROC curve and calibration curves. Time-dependent ROC curve for the radiomic signature predicting 3-year **(A)** PFS and 18-month **(B)** PFS in the WCSUH-SCU training cohort, WCSUH-SCU internal validation cohort, and the HNPPH independent external validation cohort. Time-dependent ROC curve for the radiomic nomogram predicting 3-year **(C)** PFS and 18-month **(D)** PFS in the training cohort compared with the predictive models based on clinical characteristics. Calibration curves of 3-year **(E)** and 18-month **(F)** time-dependent ROC curve of radiomic nomogram and radiomic signature. ROC curve, receiver operating characteristic curve; WCSUH-SCU, West China Second University Hospital of Sichuan University; HNPPH, Henan Provincial People's Hospital. PFS, Progress Free Survival.

Statistically significant discrimination between the PFS for the high-risk and low-risk recurrence groups, divided by median radiomic signature of the TC, was observed. Log-rank test *p*-values were *P* = 0.001, *P* < 0.001, and *P* < 0.001 for the TC, IVC, and IEVC, respectively (Figure 3). In the univariable Cox analysis, the C-indices of the radiomic signature were 0.758 (95% CI, 0.660–0.856), 0.752 (95% CI, 0.718–0.787), and 0.739 (95% CI, 0.698–0.780) for TC, ICV, and IEVC, respectively. The discrimination accuracy of the radiomic signature for predicting 3-year recurrence risk was 83.4% (95% CI, 77.3–89.6%), 82.0% (95% CI, 78.9–85.1%), and 70.0% (95% CI, 63.6–76.4%) in the TC, IVC and IEVC, respectively (Figure 4A). The discrimination accuracy of the radiomic signature for predicting 18-month recurrence risk was 82.4% (95% CI, 77.8–87.0%), 77.3% (95% CI, 74.4–80.2%), and 79.7% (95% CI, 73.8–85.6%) in the TC, IVC, and IEVC, respectively (Figure 4B).

**Figure 5:**
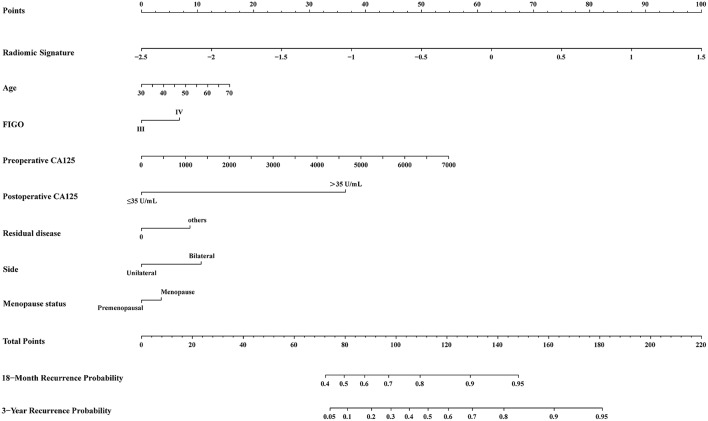
Radiomic nomogram. Probability of 3-year and 18-month progress-free survival (PFS) in patients with advanced high-grade serous ovarian cancer using the radiomic nomogram prediction model, which was developed in a training cohort with radiomic signature and seven clinical characteristics. First, locate the radiomic signature value of a patient on the *Radiomic Signature* axis and draw a line straight upward to the *Points* axis. Second, repeat the process for each variable. Third, Sum the points of the eight risk factors. Finally, locate the final sum on the *Total Point* axis and draw a line straight down to find the probability of 3-year and 18-month PFS. FIGO, International Federation of Gynecology and Obstetrics; CA-125, Carbohydrate Antigen 125.

A multivariable Cox analysis using eight independent predictors was used to develop a novel radiomic nomogram to predict the probability of recurrence within 3 years or 18 months (Figure 5; Table 5). The discrimination accuracy of the radiomic nomogram for predicting 3-year recurrence risk was 88.9% (95% CI, 85.8–92.5%) in the TC but only 73.7% (95% CI, 69.4–78.1%) via the clinical prognostic model alone (DeLong's test *P* = 0.031, Figure 4C). The discrimination accuracy of the radiomic nomogram for predicting 18-month recurrence risk was 84.1% (95% CI, 80.5–87.7%) in the TC but only 64.9% (95% CI, 59.0–70.8%) via the clinical prognostic model alone (DeLong's test *P* = 0.006, Figure 4D). The models also demonstrated favorable calibration. The *p*-values via the Hosmer-Lemeshow test for 3-year, and 18-month PFS predictive ability of the radiomic signature and radiomic nomogram were 0.199, 0.178 (Figure 4E), and 0.766, 0.839 (Figure 4F), respectively.

**Table 5:** Eight variables' coefficients of nomogram.

**Variables**	**Coefficients**
Radiomic signature	0.4858
Age	0.0076
FIGO	0.1314
Preoperative CA-125	0.0002
Postoperative CA-125	0.7080
Residual	0.1682
Tumor side	0.2069
Menopause status	0.0685

## Discussion

In the present retrospective multicenter study, we employed a radiomic analysis approach using preoperative contrast enhanced CT images data to develop a radiomic model via TC. We then built a radiomic signature for the TC, IVC, and IEVC using this model and tested its prognostic utility for advanced HGSOC (FIGO stage III or IV). We also investigated the relationship between the PFS and radiomic signature in advanced HGSOC via Kaplan-Meier survival analysis and the Cox proportional hazards model. We also implemented internal validation and independent external validation across two key time points. Finally, we developed a novel nomogram to further improve the predictive ability and verify the validity of the radiomics approach.

Our previous radiomic analysis work had been successfully applied in many different oncological diseases including HGSOC (11, 16, 17, 24, 25). Existing research has clarified the relationship between radiomic features and tumor prognosis (15, 26, 27). Quantitative radiomic features were also proposed to explain tumor characteristics and were significantly associated with patients' prognoses (13). These features were also found to have the capability to mine prognostic information from CT images that were not recognizable by eye (28). Radiomic feature data might allow for the excavation of otherwise unavailable prognostic information from CT images. For instance, radiomic analysis was found to be feasible for use in preoperative non-invasive prediction of prognosis based on CT images (10). Due to the high heterogeneity among HGSOC patients, predicting progression risk is challenging (29). However, radiomic analysis might allow for an additional method by which tumor heterogeneity can be characterized (9). Furthermore, CT provides a low-cost and non-intrusive means with which to assess tumors. Given this, distinct radiomic signature might guide clinical practice, as other clinical variables such as CA-125 level, FIGO stage, etc.

In the present study, we extracted a total of 620 3-D radiomic features. The features with interpretability were different from deep learning features (30, 31), which were generally lacking in interpretability. We calculated the intra-class correlation coefficient (ICC) of the radiomic features using the ROI selected by the two radiologists. The ICC values (range, 0.84–0.97) showed that the features were stable between two radiologists. LASSO regression analysis is a highly-dimensional, high-performance data processing algorithm commonly used in machine learning (27). We used this approach in the present study to select four radiomic features that were closely related to recurrence risk and to build a radiomic signature. Although the CT images in this study were acquired with four scanners at two different institution and with different imaging protocols, we found little impact of this variability on the prognostic model's predictivity validity, as verified in IVC and IEVC. Rather than randomly dividing patients, we grouped patients from WCSUH-SCU by their surgical timing (early and late). This revealed that our TC-built model which was built by the data of existing patients could be used to predict prognoses of newly patients. This design increased the generalization ability of the prognostic model. Median radiomic signature of TC stratified patients into high-risk and low-risk recurrence groups in each cohort. These two groups also had significantly different PFS. The prediction results for 3-year and18-month PFS revealed that CT-based radiomic analysis successfully stratified patients according to their radiomic signature values. The radiomic nomogram (incorporating both radiomic signature and seven clinical characteristics) outperformed the clinical prognostic model. Although common clinical variables can be used to predict the recurrence of ovarian cancer (32–34), predicting the recurrence of advanced HGSOC is less effective. Therefore, use of the radiomics (radiomic signature and radiomic nomogram) not only allows for the prediction of advanced HGSOC recurrence, but also complements existing ovarian cancer prognostic markers.

It is a meaningful research for accurate prediction of individual patient outcomes by means of applying radiomics approach to the analysis of advanced HGSOC. There are few studies of CT-based analysis of PFS in ovarian cancer, especially in advanced HGSOC patients. The present study not only validated our previous results but also confirmed the value of radiomics approach in better understanding tumor prognosis. Additionally, high recurrence risk might be identified using this technique in advanced HGSOC patients such that more intensive treatments might be administered. This additional information might affect the selection of chemotherapy drugs and the determination of chemotherapy regimens (35). Meanwhile, in those with elevated recurrence risk, follow-up periods might be shortened. Thus, this additional method of risk identification in HGSOC patients may have a positive impact on improving their treatment and prolonging their survival.

While the present study offers significant benefits, it also has some limitations which warrant discussion. First, it was a retrospective study with a relatively small sample size. Furthermore, all samples were collected from patients in developing countries and of the same race, limiting the applicability of the present study to more heterogeneous populations. A larger, prospective clinical trial is thus required to address these limitations. Additionally, given our use of an immature automatic segmentation algorithm, manual segmentation was used in this study. Manual segmentation may have resulted in inconsistent, subjective tumor segmentation, thereby reducing the model's performance. Based on our previous findings, further studies of automatic segmentation algorithms are required to address this limitation (36, 37).

In conclusion, radiomic signature, and radiomic nomogram may allow for the prediction of postoperative advanced HGSOC recurrence. These methods, which can be employed both before or during the perioperative period and are low-cost and non-invasive, are likely to affect clinical treatments and follow-up planning. Our results using the prognostic model suggest that radiomic signature is a potential prognostic marker and predictor of individual differences in advanced HGSOC progression.

## Ethics Statement

This study was carried out in accordance with the recommendations of the ethics committee of West China Second University Hospital of Sichuan University and the ethics committee of Henan Provincial People's Hospital with written informed consent from all subjects. All subjects gave written informed consent in accordance with the Declaration of Helsinki. The protocol was approved by the ethics committee of West China Second University Hospital of Sichuan University and the ethics committee of Henan Provincial People's Hospital.

## Author Contributions

WW (1st author), ZL, YR, MW, YG, and JT conceived of and designed the study. WW (1st author), ZL, YR, MW, YG, JT, BZ, YB, and WW (6th author) collected and assembled all data. WW (1st author) and SW analyzed and interpreted all data. WW (1st author), ZL, and YR wrote the manuscript. All authors approved of the final manuscript.

### Conflict of Interest Statement

The authors declare that the research was conducted in the absence of any commercial or financial relationships that could be construed as a potential conflict of interest.
